# Lipid metabolism is associated with developmental epigenetic programming

**DOI:** 10.1038/srep34857

**Published:** 2016-10-07

**Authors:** Elizabeth H. Marchlewicz, Dana C. Dolinoy, Lu Tang, Samantha Milewski, Tamara R. Jones, Jaclyn M. Goodrich, Tanu Soni, Steven E. Domino, Peter X. K. Song, Charles F. Burant, Vasantha Padmanabhan

**Affiliations:** 1 Department of Environmental Health Sciences, University of Michigan School of Public Health, Ann Arbor, Michigan, 48109, USA; 2 Department of Nutritional Sciences, University of Michigan School of Public Health, Ann Arbor, Michigan, 48109, USA; 3 Reproductive Sciences Program, Department of Obstetrics and Gynecology, University of Michigan Medical School, Ann Arbor, Michigan, 48109, USA; 4 Department of Biostatistics, University of Michigan School of Public Health, Ann Arbor, Michigan, 48109, USA; 5 Department of Pediatrics, University of Michigan Medical School, Ann Arbor, Michigan, 48109, USA; 6 Michigan Regional Comprehensive Metabolomics Resource Core, University of Michigan, Ann Arbor, Michigan, 48109, USA; 7 Department of Obstetrics and Gynecology, University of Michigan Medical School, Ann Arbor, Michigan, 48109, USA; 8 Department of Internal Medicine, University of Michigan Medical School, Ann Arbor, Michigan, 48109, USA; 9 Department of Molecular and Integrative Physiology, University of Michigan Medical School, Ann Arbor, Michigan, 48109, USA

## Abstract

Maternal diet and metabolism impact fetal development. Epigenetic reprogramming facilitates fetal adaptation to these *in utero* cues. To determine if maternal metabolite levels impact infant DNA methylation globally and at growth and development genes, we followed a clinical birth cohort of 40 mother-infant dyads. Targeted metabolomics and quantitative DNA methylation were analyzed in 1st trimester maternal plasma (M1) and delivery maternal plasma (M2) as well as infant umbilical cord blood plasma (CB). We found very long chain fatty acids, medium chain acylcarnitines, and histidine were: (1) stable in maternal plasma from pregnancy to delivery, (2) significantly correlated between M1, M2, and CB, and (3) in the top 10% of maternal metabolites correlating with infant DNA methylation, suggesting maternal metabolites associated with infant DNA methylation are tightly controlled. Global DNA methylation was highly correlated across M1, M2, and CB. Thus, circulating maternal lipids are associated with developmental epigenetic programming, which in turn may impact lifelong health and disease risk. Further studies are required to determine the causal link between maternal plasma lipids and infant DNA methylation patterns.

Fetal development is a sensitive window for epigenetic reprogramming that can have lifelong impacts on gene expression and subsequent disease risk. Demethylation and subsequent reprogramming of epigenetic marks occurs in the mammalian preimplantation embryo and developing fetus^1^. Increasing evidence shows that maternal nutrition impacts uterine metabolic environment and successive fetal epigenetic programming^2^,^3^,^4^. For example, studies in animal models demonstrate that maternal diet modulation, including caloric^5^,^6^ and protein restriction^7^,^8^, over-nutrition^9^,^10^, and micronutrient supplementation^11^, alters DNA methylation at candidate gene loci and globally at cytosine-guanine (CpG) dinucleotides and repetitive elements, including long interspersed element 1 (LINE1).

Plasma metabolite levels provide a more objective measure of the maternal environment to which the developing fetus is exposed, compared to self-reported dietary data. The metabolome is relatively stable over time in non-pregnant adults^12^,^13^,^14^, and can be used as a proxy for dietary behavior of individuals interacting with genetic factors^15^,^16^. In contrast, circulating nutrient levels vary across pregnancy depending on the stage of fetal growth. In the first trimester of pregnancy, increased adipose tissue response to insulin results in anabolic storage of maternal fatty acids; in the third trimester fetal nutrient demands intensify, driving a catabolic release of maternal lipid stores and a subsequent rise in maternal plasma lipid levels^17^,^18^,^19^. Despite third trimester catabolism, fetal demand for long chain polyunsaturated fatty acids (LCPUFA) overwhelms maternal desaturase activity^20^; thus, maternal dietary intake of LCPUFA is critical for maintenance of adequate fetal levels^21^,^22^. The use of metabolomics to characterize maternal metabolites across pregnancy is only beginning to be realized^23^,^24^.

This study investigates the changes in the maternal metabolome and epigenomic state from pregnancy to delivery and the extent to which maternal states compare to those of their infants at birth. The impact of the maternal metabolites on the epigenetic patterning in offspring is likely dependent on the timing, duration, and magnitude of exposure. We performed targeted metabolomics profiling of 40 mother-infant dyads in **1**^**st**^ **trimester maternal plasma (M1)** as well as **delivery maternal plasma (M2)** and infant umbilical **cord blood plasma (CB)**. We then correlated metabolites to global and candidate loci DNA methylation profiles of the maternal and fetal peripheral blood cells to address the following questions: 1) Do maternal metabolomics profiles change from trimester 1 to delivery? 2) Do cord blood metabolome and epigenome patterns mirror those of 1^st^ trimester and term blood? 3) Are specific metabolites associated with DNA methylation status in cord blood? The results suggest a relationship between maternal lipid-derived metabolites and cord blood DNA methylation status, which may lead to the identification of predictive, and modifiable, biomarkers of epigenetic programming of offspring.

## Results

### Maternal-infant characteristics

The 40 women included in this study were predominantly white (85%), non-Hispanic (95%), married (80%), and non-smokers (92.5%), with ages ranging from 21–42. All infants included in the study were from full-term pregnancies (>37 weeks), most were delivered vaginally (85.0%), no babies were classified as low birth weight (<2,500 g), and 50% were female. The majority of moms had Trimester 1 body mass index (BMI) in the healthy or overweight range (80%); maternal BMI was not correlated with pregnancy weight gain (p = 0.798), but tended to suggest an association with infant birth weight (p = 0.073). Additional demographic and physiologic measures are presented in Table 1.

### Maternal metabolomics and epigenetic profiles from pregnancy to delivery

**Figure 1:**
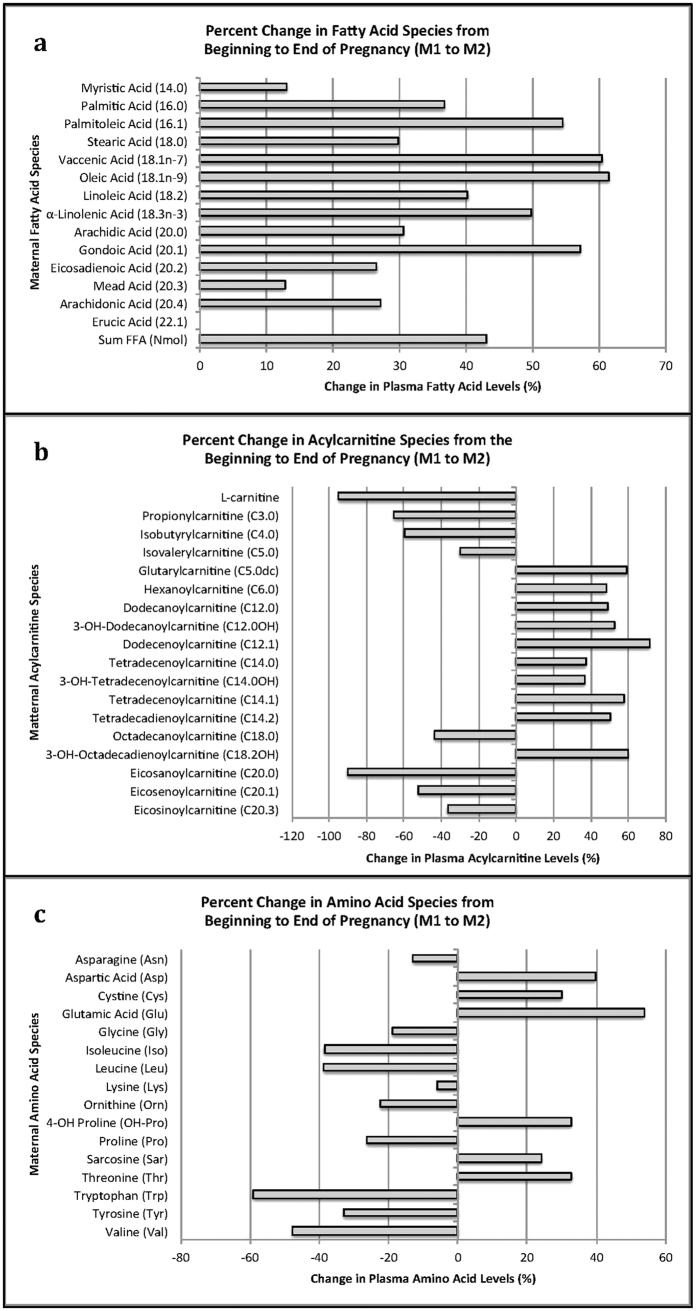
Maternal Metabolites that Changed Significantly from Trimester 1 (M1) to Delivery (M2). Forty-six of the 76 (59.5%) metabolites measured changed significantly from Trimester 1 (M1) to Delivery (M2). For all three graphs: (**A**) free fatty acids, (**B**) acylcarnitines, and (**C**) amino acids, bars to the right of zero illustrate metabolites that increased across pregnancy; bars to the left of zero show metabolites that decreased across pregnancy.

**Figure 2:**
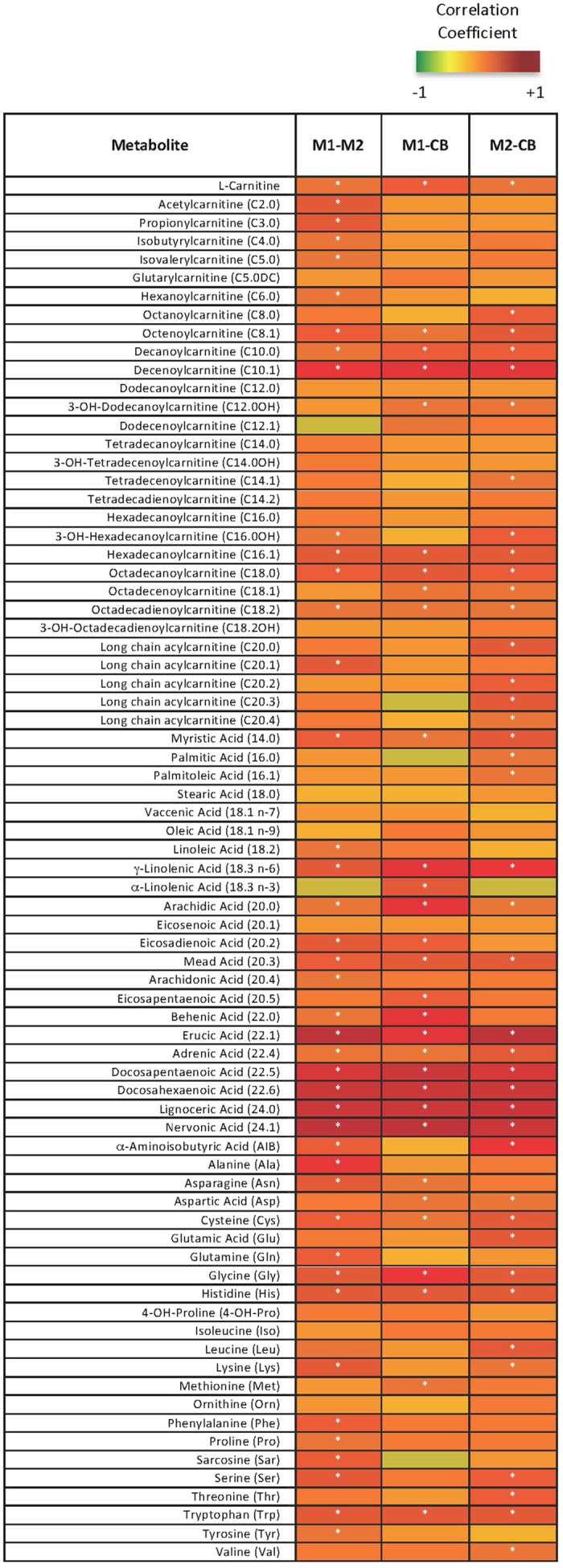
Significant Correlations between Maternal Metabolites at M1 and M2 and Infant Delivery Cord Blood (CB) Metabolites. Pearson’s Correlations were performed on metabolite data and plotted into a heatmap. The darker red boxes indicate a larger positive correlation coefficient; smaller and negative coefficients are yellow to green. Significant correlations (adjusted p < 0.05) are denoted with a white asterisk.

Plasma long-chain fatty acids (LCFA) and corresponding long-chain acylcarnitines (LCAC) increased (p < 0.001) by 1.4–2.6 fold (Fig. 1A,B, Table S1) from M1 to M2, consistent with the known increase in maternal fatty acid concentration and utilization, thought to spare glucose and amino acid (AA) use for the fetus^25^. In contrast, branched chain amino acids (BCAA) and short chain acylcarnitines (SCACs) decreased (p < 0.001) by 0.6–0.7 fold (Fig. 1B,C) from M1 to M2, consistent with an increase in nonoxidative utilization of BCAA later in pregnancy^26^. Forty-two of the 75 measured metabolites, including essential fatty acids (EFA, linoleic acid (LA) and α-linolenic acid (ALA)) and conditionally essential fatty acids (CEFA, derived from LA and ALA), LCACs, and AAs were significantly correlated (adjusted p < 0.05) in mothers from M1 to M2 (Fig. 2, Table S2).

**Figure 3:**
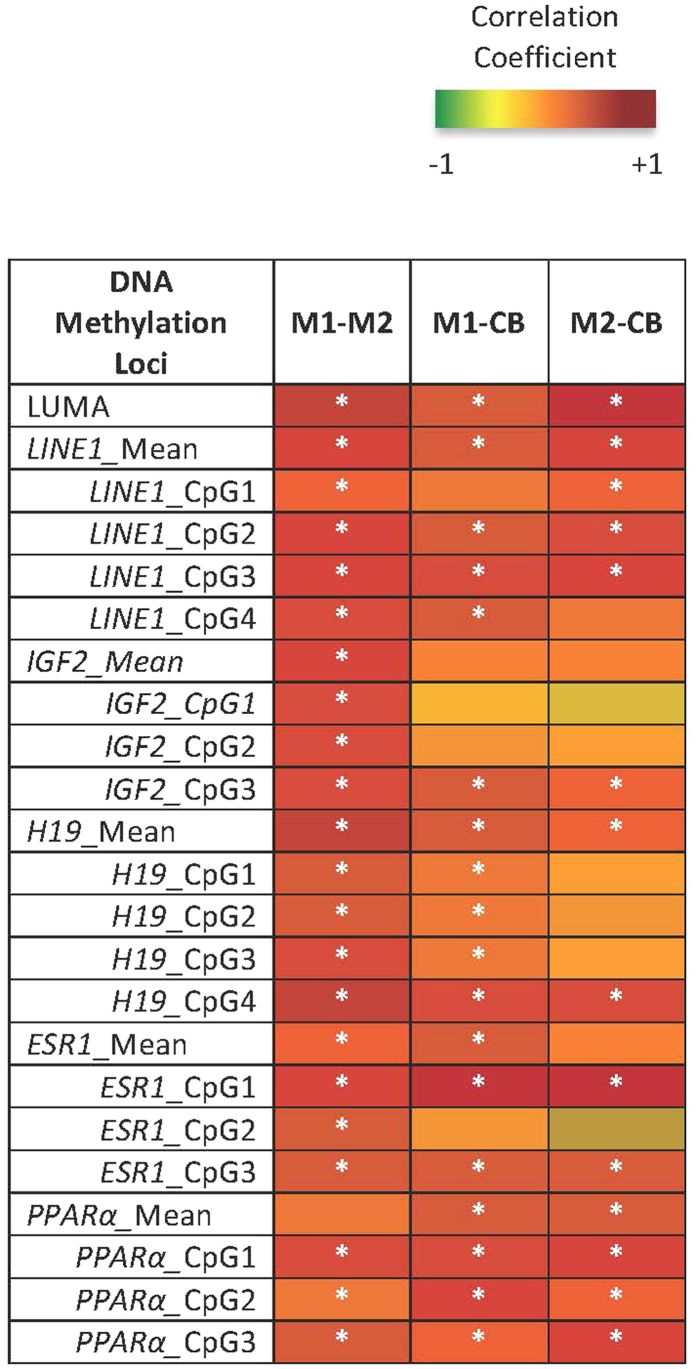
Correlations between Maternal DNA Methylation at M1 and M2 and Infant Delivery Cord Blood (CB) Methylation. Pearson’s Correlations were performed on global (LUMA, LINE1) and candidate gene (*IGF2, H19, ESR1*, and *PPAR alpha*) methylation data and plotted into a heatmap. The darker red boxes indicate a larger positive correlation coefficient, smaller and negative coefficients are yellow to green. Significant correlations (p < 0.05) are denoted with a white asterisk.

Maternal global DNA methylation levels increased slightly, by 1.02 fold (1.86%, p = 0.010), from MI to M2, as measured via the Luminometric Methylation Assay (LUMA) at CCGG sites throughout the genome. Mean DNA methylation at maternal Estrogen Receptor 1 (*ESR1*) decreased by 0.83 fold (−19.83%, p = 0.001) from M1 to M2 (Table S3). Methylation at LINE1, Peroxisome Proliferator-Activated Receptor Alpha (*PPARα*) and the imprinted loci Insulin-Like Growth Factor-2 (*IGF2*) and *H19*, was unchanged across pregnancy. Whether or not there was a change over time, the levels of methylation were significantly correlated (adjusted p < 0.05) between M1 and M2 (Fig. 3, Table S4).

### Infant metabolome and methylome mirror their mothers

Of the 75 metabolites measured, levels of 24 metabolites were positively correlated (adjusted p < 0.05) between infant (CB) and maternal plasma at both M1 and M2. (Fig. 2, Table S2). These include the EFA critical for brain development (18:3 (n-6))^17^ and the conditionally essential derivatives (20:3, 22:4, 22:5, 22:6)^27^,^28^. Forty-six metabolites significantly correlated between mothers at either time point (M1 or M2) and infants (CB); 23 (50%) of these were also stable in maternal plasma from M1 to M2, as reported above. This consistency suggests tight regulation of these metabolites, especially those involved in fatty acid biosynthesis and elongation pathways, during pregnancy.

DNA methylation levels at LUMA, *LINE1*, and *PPARα* were positively correlated (adjusted p < 0.001, p < 0.05, and p < 0.05, respectively) within maternal-infant dyads (M1, M2, CB) (Fig. 3, Table S4). M1 *H19* DNA methylation positively correlated (adjusted p < 0.05) with both M2 and CB. However, M1 *IGF2* DNA methylation was significantly correlated (p < 0.05) only with M2, not CB. Significant correlations between maternal and infant DNA methylation were CpG site specific within *ESR1* (sites 1 and 3, p < 0.05) (Table S4). Percent DNA methylation did not differ by infant sex at any CpG sites assessed.

### Metabolites associated with DNA methylation status in infant cord blood leukocytes

The correlation among maternal metabolites and infant DNA methylation depended on genomic region. At both M1 and M2, there was a significant correlation (p < 0.05) of EFAs, CEFAs, medium chain acylcarnitines (MCAC) and LCACs with DNA methylation levels and at LUMA, LINE1*, ESR1*, and *PPARα* sites, although the direction differed – with positive correlations at LINE1, *ESR1*, and *PPARa* and a negative correlation at LUMA. At M1, there were fewer significant correlations between metabolites and imprinted genes, *IGF2* and *H19*, compared to non-imprinted genes and measures of global methylation. At M1, the LCFA 20:3 and the LCAC C20:4 were significantly correlated (p < 0.05) to methylation of the imprinted gene *IGF2*; by M2, the EFA linoleic acid and CEFAs (20.1, 20.3, 20.4) were significantly correlated (p < 0.05). In contrast, AAs were most highly correlated with the imprinted gene *H19*, but only valine was significant (p < 0.05) at M1 (Table 2, Figure S2).

**Figure 4:**
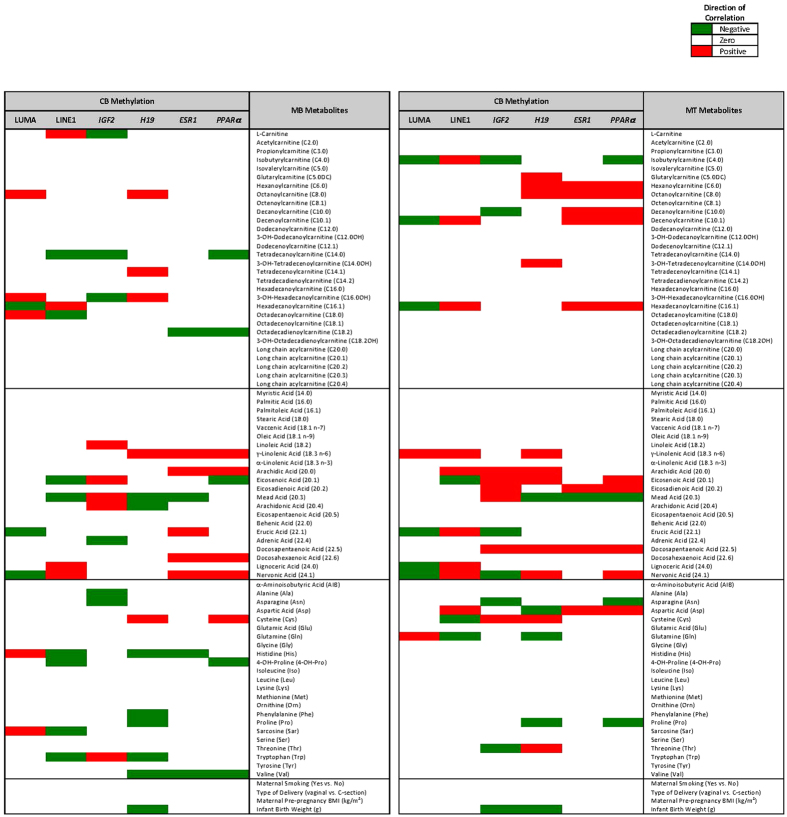
Multivariate Regression Analyses Illustrating the Maternal Metabolites Associated with Cord Blood DNA Methylation. Multivariate regression was used to investigate the joint associations between all metabolites and mean DNA methylation, adjusting for confounders: maternal smoking, maternal prepregnancy BMI, delivery mode, and infant birth weight. M1 and M2 metabolites that were significantly associated with global or candidate gene loci are plotted in the heatmap. Negative associations are denoted in blue, positive associations in red.

Certain maternal metabolites were commonly correlated to infant DNA methylation at many genic loci, when ranked by p-value (Table 2). Nervonic acid (24:1) and decenoylcarnitine (C10:1) were the top metabolites, significantly correlating to 8/12 and 7/12 gene loci at M1 (p < 0.05) or M2 (p < 0.05), followed by DHA (22:6, 6/12), erucic acid, DPA and lignoceric acid (22:1, 22:5 & 24:0, 5/12) supporting the premise that LCFAs derived from EFAs could play a role in modulation of the infant epigenome. When multivariate regression analyses were performed (Fig. 4), EFA and CEFAs associated significantly with CB methylation. Many of the same M1 and M2 metabolites significantly associated with changes in global methylation at both LUMA and LINE1 sites (Fig. 4, left columns); however, the direction of association differed (e.g. nervonic acid is negatively associated with LUMA methylation, but positively associated with LINE1 methylation). Because the two global methylation measures represent different aspects of global methylation (LUMA assesses methylation at every CCGG region and LINE1, a specific repetitive element), the different outcomes are not surprising, although it emphasizes the need to choose a global methylation measure that is relevant for each study. Lastly, of the potential confounding variables included in the regression models (maternal smoking, pre-pregnancy BMI, delivery mode, and infant birth weight), only infant birth weight was significantly associated with DNA methylation and only at imprinted gene loci (Fig. 4, middle columns). Methylation status of *IGF2* and *H19* is related to fetal growth^29^, so an affect on birth weight is expected.

Significant correlations between CB metabolites and CB DNA methylation followed similar trends to those of maternal metabolites and CB DNA methylation correlations (Table 2). For example, CB CEFAs negatively correlated (p < 0.001) with CB LUMA methylation and positively correlated (p < 0.001) at LINE1 and *ESR1* sites. The few CB metabolites that significantly correlated (p < 0.05) with CB *IGF2* and *H19* were amino acids (His, Trp, and Cys). No CB metabolites appeared to relate to *PPARα* methylation.

## Discussion

This is the first study in human subjects to investigate the role of the maternal metabolic environment in fetal epigenetic reprogramming. Most human epidemiologic birth cohorts rely on maternal diet as an indicator of metabolic environment^30^,^31^,^32^. However, compared to maternal self-reported dietary data, examining maternal metabolomics profiles provides a more precise, non-biased, and comprehensive measure of the metabolic environment to which a developing fetus is exposed. The study design involving maternal sampling from the same subject dyads during first trimester and delivery also provided an opportunity to investigate whether the timing and duration (from M1 to M2) of maternal exposure impact infant DNA methylation. This study design revealed the following novel findings that very long chain fatty acids (22:1, 22:5, 22:6, 24:0, 24:1), medium chain acylcarnitines (C10.1 and C16.1), and histidine were: (1) stable in maternal plasma across pregnancy, (2) significantly correlated between maternal and infant plasma, and (3) in the top 10% of maternal metabolites correlated with infant DNA methylation. This suggests that these highly regulated metabolites may have a potential role in determining the levels of DNA methylation in the offspring.

Regarding maternal metabolite changes across pregnancy, we found higher circulating LCFA (16 s–18 s) levels, including EFAs, at M2 compared to M1, but no change in circulating VLCFAs (20 s–24 s), MCAC (C10.1), LCACs (C16-C20), or AAs. Traditionally, maternal lipolysis coupled with a decrease in maternal lipoprotein lipase activity and a metabolic state similar to starvation have characterized the third trimester of pregnancy^33^,^34^. The higher circulating LCFAs at M2 vs. M1 we observed are consistent with this established trend, supporting the theory that maternal third trimester hypertriglyceridemia fuels the rapid fetal growth characteristic of this trimester^33^,^34^. Consistent with the increase in the levels of LCFA oxidation is the increased levels of MCAC. Interestingly, while the levels of the acylcarnitines derived from the 2 major fatty acid species, C16:0 and C18:1 were unchanged the levels of the VLCAC derived from LA and ALA (C20:3 and C20:4) were reduced. We speculate that this may be due to diversion of these CEFA into biosynthetic pathways.

The conservation of many VLCFAs, MCAC, LCACs and AA levels in maternal plasma across pregnancy is a novel finding. This may occur to support healthy fetal neurodevelopment. Fatty Acid Desaturase 1 and 2 (*FADS1*, *FADS2*) encode delta-5 and delta-6 desaturases, which are the rate-limiting steps in the conversion of LA and ALA to the longer, more unsaturated and biologically active EPA, DHA and arachidonic acid; *FADS1* and *FADS2* are also related to levels of VLCFA in blood cells and plasma^35^. Fetal delta-5 and delta-6 desaturase activity are negligible prior to birth, so fetal growth is dependent on placental transfer of LCPUFA^21^. Thus, maternal dietary intake of LCPUFA is critical to achieve adequate fetal levels^20^,^22^, suggesting maternal diet may drive the fetal programming effect observed as differential DNA methylation in cord blood at birth.

Comparing maternal and infant plasma metabolite levels also demonstrated highly correlated levels of MCACs (C8 s, C10 s), LCACs (C16 s, C18`s), EFA (X18.3 n-6), VLCFA (X22 s, X24 s), and certain AAs (Cys, Gly, His, Trp). The fact that these are the same metabolites that were highly conserved from M1 to M2 further supports the developmental importance of lipid metabolism. Previous studies have reported fetal LCFA levels depend on circulating maternal LCFAs^17^,^36^, and observed preferential transport of DHA from maternal plasma to placental and fetal tissues occurs in the third trimester of pregnancy^37^.

Thus, maternal regulation of lipid metabolism during pregnancy is likely critical for fetal health. VLCFAs, especially DHA (22.6) and nervonic acid (24.1), are vital components of myelin sheaths; adequate levels of these VLCFAs stimulate neuronal growth^27^,^28^,^36^,^38^. VLCFAs are likely important components of fetal neurodevelopment, and thus maternal-fetal levels of these metabolites may be tightly regulated across pregnancy to ensure fetal health.

Surprisingly, the comparison of maternal-infant epigenomes revealed similarities in global and candidate gene DNA methylation levels between the dyads. Erasure of parental DNA methylation marks in mammals occurs early in the first trimester, prior to implantation^39^. Fetal epigenetic reprogramming follows in two waves (1) in preimplantation zygotes during early embryogenesis and (2) in primordial germ cells (PGC) of the developing fetus, differentially by offspring sex^40^,^41^,^42^. Repetitive elements, including LINE1, are known to remain highly methylated in PGCs^42^,^43^ and preimplantation embryos^43^, suggestive of the need to repress transposon movement in order to maintain genomic stability. The significant correlation between maternal-infant LUMA, LINE1 and candidate gene methylation, despite global reprogramming in early development^39^, implies that methylation status at some CpG sites may be influenced by maternal genetics and/or shared environment.

The maternal metabolites that were significantly correlated to infant DNA methylation, largely overlapped the metabolites that remained constant from M1 to M2 and the metabolites that correlated between mother-infant pairs, signifying a potential role in epigenetic programming. The observed positive correlation of VLCFAs with methylation at LINE1 retrotransposons and in the promoter regions of candidate genes (*ESR1, PPARα*) is supported by previous findings that DHA exposure affects DNA methylation globally and at metabolic and inflammatory candidate gene loci in *in vitro*^44^, murine^45^, and human^46^,^47^,^48^ studies. In the Southampton Women’s Survey birth cohort, maternal third trimester plasma total n-6 PUFAs positively associated with child adipose mass while total n-3 PUFAs positively correlated with lean mass at four and six years of age, suggestive of age-specific metabolic reprogramming effects of prenatal PUFA exposure during human fetal development^49^.

Our study also found that infant *H19* methylation correlated most strongly with maternal amino acid metabolites indicative of maternal protein playing a critical role in DNA methylation patterns at imprinted gene loci. Imprinted genes maintain allele-specific epigenetic marks based on the parent of origin of the transmitted allele^50^. Altered DNA methylation and gene expression of imprinted *IGF2* and *H19* loci in offspring following perinatal protein restricted diets are well documented in animals^51^,^52^,^53^ and humans^54^. Hypermethylation at *H19* positively correlates to weight-for-age greater than 85^th^ percentile in infants^55^ and greater skinfold thickness measures and subcutaneous adiposity in teens^56^, demonstrating metabolic effects on offspring from circulating maternal amino acids in addition to circulating VLCFAs.

These study findings provide important insight into understanding the sensitivity of the fetal epigenome to maternal metabolic environment. Additional studies are needed, however, to further characterize the maternal-fetal metabolic milieu in a larger sample size, with more frequent time-points throughout pregnancy and with longitudinal follow-up of infants into childhood to assess potential alterations in growth and development resulting from differential epigenetic reprogramming. Maternal blood collection at delivery may not be representative of the metabolic and epigenomic states during the third trimester due to the stress of parturition; collection earlier in the third trimester would improve characterization of the changing metabolic environment. The investigation of other epigenetic mechanisms (e.g. histone modifications, miRNAs) would also provide a more comprehensive evaluation of pregnancy-related impacts on fetal programming.

## Conclusion

Understanding the role of maternal metabolomics across pregnancy and the impact of metabolites on fetal epigenetic programming in healthy moms with uncomplicated pregnancies, as examined in this study, provides a point of comparison for the numerous studies now being conducted on the impact of maternal conditions, such as obesity, gestational diabetes, and non-alcoholic fatty liver disease, on fetal metabolic programming and birth outcomes. The study design allowed for the novel comparison of metabolites and epigenetic marks from pregnancy to delivery in the mother and the comparison of maternal measures to infant levels. Significant findings include the tight regulation of LC/VLCFA and MC/LCAC levels in maternal blood across pregnancy and in cord blood at delivery. The fact that the same LC/VLCFAs, MC/LCACs, and histidine correlate with infant cord blood DNA methylation is noteworthy since these maternal metabolites have previously been associated with fetal neuro-visual development (LC/VLCFA) and general fetal growth (histidine and other amino acids). DNA methylation at CCGG sequences, LINE1 repetitive elements and some genic loci were highly correlated between maternal blood and infant cord blood. Thus, the shared genetic and/or environmental factors of mother-infant dyads impact DNA methylation, potentially contributing to the developmental origins of health and disease risk. This study is the first to examine the impact of individual plasma metabolites on infant DNA methylation globally and in candidate imprinted and non-imprinted genes, which provided more detailed insights into fetal responses to the maternal metabolic milieu. Once fully characterized, a metabolomics assessment from maternal blood drawn during prenatal clinic visits may serve as useful screens for fetal nutritional sufficiency and subsequent disease risk, thus providing unprecedented benefit for early preventive care and lifestyle recommendations to decrease disease incidence beginning at birth.

## Materials and Methods

### Subject Population

**Figure 5:**
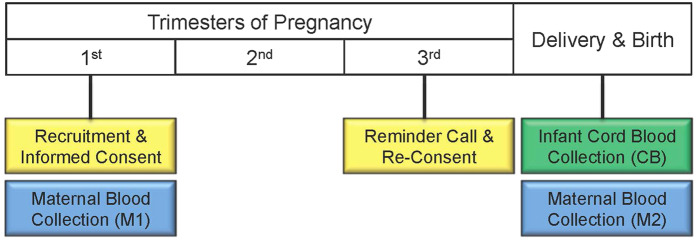
Timeline of Clinical Study Visits. Mothers were recruited, consented, and blood was drawn during a 1^st^ trimester prenatal clinic visit. Maternal blood was also drawn when women were admitted to the hospital, prior to delivery. Infant cord blood was collected immediately after delivery.

Forty women were recruited from the community surrounding the University of Michigan Von Voigtlander Women’s Hospital, after review and approval by the University of Michigan Institutional Review Board (IRBMED: HUM00017941). The study was carried out in accordance with the approved guidelines and regulations, including appropriate informed consent. Women were contacted during their 1^st^ trimester prenatal clinic visit and were included in the study if they fit the following inclusion criteria: 18 years or older, natural conception (no infertility treatment), singleton pregnancy, 8 to 14 weeks pregnant (Fig. 5). At this initial study visit, participants had blood drawn via venipuncture and filled out a simple, one-page study questionnaire. The questionnaire included items on race, ethnicity, income, and smoking status. Women were re-contacted between 34 to 38 weeks of pregnancy and provided study materials to ensure they understood sample collection procedures during delivery. Upon admission for delivery, venous blood samples were drawn. After delivery of the placenta, cord blood samples were collected via venipuncture from the umbilical cord, which was clamped proximal to the placenta. Due to limited availability of biological samples, epigenetic and metabolomics analyses were performed on a subset of the maternal-infant dyads: M1-M2 n = 37, M1-CB n = 32, M2-CB n = 32, CB-CB n = 33.

### Mass-Spectrometry Based Metabolomics Analysis

Directed metabolomics analyses (76 metabolites) were conducted on M1, M2, and CB plasma samples. Twenty-three amino acids (AA) and 23 free fatty acids (FFA) were measured via gas chromatography-mass spectrometry (GC-MS) on an Agilent 7890A-5975C instrument. Agilent CHEMSTATION software was used to analyze the MS spectral data. Thirty acyl carnitines (AC) were measured via reverse-phase liquid chromatography-mass spectrometry (LC-MS) on an Agilent 1200 LC/6400 series triple quadrupole mass spectrometer with electrospray ionization source (ESI), which is operated in positive mode. Data were processed by MassHunter Quantitative software; metabolite levels are presented as absolute measures, not relative amounts. Rationale and quality control measures for metabolomics analyses are explained in SI Materials and Methods.

### DNA Methylation Analysis

Fifty ng of bisulfite converted DNA (SI Materials and Methods) from each sample was used in each PCR reaction mixed with HotStarTaq master mix (Qiagen), 10 pmol forward primer and 5 pmol biotin-labeled reverse primer followed by assay specific PCR cycling parameters. Primer sequences and PCR cycling parameters for the following assays are provided in the Supporting Information (LINE1*, IGF2, H19, ESR1, PPARα*). Products amplified by PCR were verified via gel electrophoresis. The PyroMark ^TM^Q96 MD pyrosequencing system (Qiagen) uses a predetermined sequence-to-analyze to dispense nucleotides. PyroMark Q-CpG software (Qiagen) computes the ratio of cytosine to thymine signal intensity at each CpG site in the assays; data outputs as percent methylation. The Luminometric Methylation Assay (LUMA) uses restriction enzymes to digest genomic DNA and detects methylation at 5′-CCGG-3′ sequences across the genome. A detailed description of LUMA analysis via pyrosequencing has been described previously^57^,^58^; briefly, global DNA methylation is calculated by the ratio of normalized product signal between methylation sensitive and insensitive digestions from the same sample: 1-[(*HpaII*/*EcoR1*)/(*MspI*/*EcoR1*)] × 100.

The Luminometric Methylation Assay (LUMA) provides a global measure of DNA methylation at CCGG sites and has been used in both clinical and longitudinal epidemiologic studies^57^. Altered global methylation assessed via LUMA has been associated with environmental exposures in humans^59^. LINE1, a measure of long interspersed retrotransposons across the genome, is another method of measuring global methylation^60^ and has been shown to be a useful marker of chemical exposure^61^. While LINE1 methylation is a convenient way to measure global methylation, gene-specific promoter regions may methylate differently in response to exposures, making it essential to measure both global and candidate genes of interest^62^.

Candidate loci were selected for investigation based on their role in fetal growth and development and previously reported associations between loci and fetal programming effects. A significant body of research has investigated the importance of imprinted genes, *IGF2* and *H19*, in healthy fetal development^63^,^64^. Imprinted genes maintain allele-specific epigenetic marks based on the parent of origin of the transmitted allele^50^. Maternal nutrient intake^53^,^55^,^65^,^66^ has been associated with alterations in methylation at the imprint control regions (ICRs) for *IGF2* and *H19*. Expression of these imprinted genes, critical for fetal development and rapid somatic growth in the early postnatal period, decreases after this initial growth phase, coordinating growth deceleration across tissues to limit adult body size^67^.

For non-imprinted genes, estrogen receptor 1 (*ESR1*) is an estradiol-sensitive signal transducer important in the progression of labor and delivery^68^ and is associated with development of obesity and non-alcoholic fatty liver disease later in life^69^,^70^,^71^. Similarly, DNA methylation of *PPARα* has been implicated as a fetal programmer of metabolic health and disease^72^. Early life exposure to leptin increases *PPARα* variant transcription in the liver; transcriptional changes are sustained into adulthood^73^. Many other fetal candidate genes may be impacted by the maternal metabolome; this study served as a proof of concept that both imprinted and non-imprinted genes could be affected. Additional studies in this human birth cohort plan to expand the candidate gene panel to investigate the impact on other gene loci. PCR parameters and PCR and pyrosequencing primers used in optimized assays to identify DNA methylation at each candidate locus are provided in Table S5.

### Statistical Analysis

Clinical data collected during the 1^st^ trimester and delivery clinic visits were combined with metabolomics and DNA methylation data into one large dataset. No outliers, defined by a cutoff of more than three standard deviations, were identified in epigenetics or metabolomics data. DNA methylation measures were normally distributed, so values are presented as mean and standard deviation (SD); paired t-tests were used to compare maternal methylation at M1 and M2. Despite no significant outliers, metabolite levels were all right skewed, so results are presented as median and interquartile range (IQR); non-parametric tests (Kendall’s rank correlation and Kruskal-Wallis tests) were used to carry out bivariate analyses. In order to correct for multiple testing and control false discovery rate (FDR), the Benjamini-Hochberg method was used to calculate adjusted p-values to determine significance of results^74^.

Multivariate regression models were fitted to simultaneously investigate the adjusted association between all metabolites and mean DNA methylation at all loci, with adjustment for confounders. Using a penalized multivariate linear regression model, coefficients of unimportant predictors and confounders are estimated to exactly zero (fitting is done using the remMap package in R^75^). The important non-zero associations from penalized regression were then plotted via heat maps based on the magnitudes of the beta coefficients. Additional details regarding bivariate analyses and false discovery rate are provided in Supporting Information. All analyses were performed in R Studio, Version 3.0.2.

## Additional Information

**How to cite this article**: Marchlewicz, E. H. *et al*. Lipid metabolism is associated with developmental epigenetic programming. *Sci. Rep*. **6**, 34857; doi: 10.1038/srep34857 (2016).

## Supplementary Material

Supplementary Information

## Figures and Tables

**Table 1:** Cohort Characteristics and Physiologic Measures (N = 40).

Cohort Characteristics	Number (n)	Percent (%)
Maternal Characteristics		
Race/Ethnicity		
Asian	0	0.0
African American	2	5.0
Hispanic	2	5.0
Native American	1	2.5
White	34	85.0
More than one race	1	2.5
Marital Status		
Single	8	20.0
Married	32	80.0
Smoking		
Never smoked	28	70.0
Quit before pregnancy	9	22.5
Current smoker	0	0.0
Missing data	3	7.5
Body Mass Index (BMI, kg/m^2^)		
<18.5	0	0.00
18.5–24.9	24	57.5
25.0–29.9	9	22.5
30.0–34.9	3	7.5
>35.0	4	10.0
Newborn Characteristics		
Newborn Gender		
Male	20	50.0
Female	20	50.0
Preterm		
Yes	0	0.0
No	40	100.0
Delivery Mode		
Vaginal	34	85.0
C-Section	6	15.0
Apgar Score		
Severely Depressed (1–3)	0	0.0
Moderately Depressed (4–6)	2	5.0
Excellent Condition (7–9)	38	95.0
Physiologic Measures	Mean (SD)	Range
Maternal Characteristics		
Age (years)	32.28 (5.06)	21–42
Pregnancy weight gain (kg)	13.69 (4.61)	6–24
Height (cm)	167.10 (6.15)	155–177
Gestational age at delivery (days)	277.43 (6.41)	262–292
Newborn Characteristics		
Birth weight (g)	3533.38 (529.50)	2630–4575

**Table 2:** Ranked Correlation Analysis of Metabolites (M1, M2, CB) and Mean Infant (CB) DNA Methylation.

Top 10% M1 Metabolites Correlated with CB Mean DNA Methylation (n = 32)						
Infant Cord Blood - Mean DNA Methylation						
Metabolite Rank	LUMA	LINE-1	*IGF2*	*H19*	*ESR1*	*PPARα*
1	C16.1*	24.1*	20.3*	Val*	20.0*	22.6*
2	22.1*	His*	C20.4*	Iso	22.6*	C20.0*
3	24.1*	24.0*	**20.4**	14.0	22.5*	24.1*
4	C10.1*	C16.1*	C10.0	Cys	18.3n-6*	**22.5**
5	C18.0*	Trp*	C14.0OH	Pro	His*	**18.3n-6**
6	24.0*	C10.1*	C10.1	Phe	220*	Val
7	His*	22.1*	C12.0OH	Leu	24.1*	C18.2
8	22.4*	C18.0*	C16.1	C14.0	C10.1*	C18.1
**Top 10% M2 Metabolites Correlated with CB Mean DNA Methylation (n = 32)**						
1	22.1*	24.0*	18.2*	18.3n-6*	C10.1*	24.1*
2	24.0*	C10.1*	Car*	C6.0*	22.5*	C10.0*
3	24.1*	22.1*	20.3*	C14.0OH*	22.6*	C12.0*
4	C10.1*	C16.1*	20.4*	Trp*	24.0*	22.5*
5	22.5*	24.1*	20.1*	C18.2OH*	24.1*	22.6*
6	22.6*	22.5*	SumFFA*	18.0*	20.3*	C8.0*
7	C16.1*	22.6*	C3.0*	Pro	C12.OH*	C10.1*
8	C18.0*	Trp*	**16.1**	**C5.0DC**	22.1*	C12.0OH*
**Top 10% CB Metabolites Correlated with CB Mean DNA Methylation (n = 33)**						
1	24.1***	C18.0***	His*	Cys*	His**	C8.1
2	22.1***	24.1***	Trp*	**C5.0**	18.3n6**	C18.0
3	C10.1***	22.5***	22.5	C20.4	C10.1**	**Sar**
4	22.5***	24.0***	**20.3**	C20.0	22.6**	C18.1
5	24.0***	22.6***	**Glu**	C20.3	22.5*	**24.1**
6	22.6***	22.1***	Sar	**20.0**	24.1*	C20.0
7	C16.1***	C10.1***	**OH-Pro**	**X18.3n6**	C18.0*	C20.3
8	Val**	His***	24.0	Trp	Sar*	**Lys**

Kendall’s Tau, non-parametric correlation analyses comparing metabolites (M1, M2, CB) to CB DNA methylation were ranked by p-value. Significant correlations are denoted by asterisks: *p < 0.05, **p < 0.01, ***p < 0.001. The metabolite with the most significant p-value is ranked as number 1. **Bold** font indicates positive correlation coefficients; standard font indicates negative correlation coefficients. SumFFA is a summation measure of the 22 free fatty acids measured in this study. To differentiate fatty acids from acylcarnitines, acylcarnitines have a ‘C’ placed in front, e.g. C10.1; whereas fatty acids begin with the number of carbons in their chain, e.g. 24.1.
