# Supplementary material for: Lipid metabolism is associated with developmental epigenetic programming
Source: Sci Rep. 2016 Oct 7;6:34857. doi: 10.1038/srep34857 (PMC5054359; doi:10.1038/srep34857)
Supplement: Supplementary Information [file srep34857-s1.pdf]

TITLE PAGE

Long Title: Lipid metabolism is associated with developmental epigenetic programming
Short Title: Lipid metabolism associates developmental programming

Authors

Elizabeth H. Marchlewicz[a], Dana C. Dolinoy[a,b,c], Lu Tang[d], Samantha Milewski[e], Tamara R. Jones[a], Jaclyn M. Goodrich[a], Tanu Soni[f], Steven E. Domino[g], Peter X.K. Song[d], Charles Burant[b,f,h], Vasantha Padmanabhan[a,b,c,e,g,h]*

Author Affiliations

[a]Department of Environmental Health Sciences, School of Public Health;
[b]Department of Nutritional Sciences, School of Public Health;
[c]Reproductive Sciences Program, Department of Obstetrics and Gynecology, University of Michigan Medical School;
[d]Department of Biostatistics, School of Public Health;
[e]Department of Pediatrics, University of Michigan Medical School;
[f]Department of Internal Medicine, University of Michigan Medical School;
[g]Department of Obstetrics and Gynecology, University of Michigan Medical School;
[h]Department of Molecular and Integrative Physiology, University of Michigan Medical School, Ann Arbor, Michigan, 48109, USA

*** Corresponding Author Information**:

Name: Vasantha Padmanabhan
Address: 7510 MSRB I
1150 West Medical Center Dr.
University of Michigan
Ann Arbor, MI 48109-5718
Phone: (734) 647-0276
Email: vasantha@umich.edu

Keywords

(1) developmental biology, (2) metabolomics, (3) epigenetics, (4) human birth cohort, (5) developmental origins of health and disease (DOHaD)

SUPPORTING INFORMATION

Materials and Methods

Sample Collection and Handling. Three types of blood collection tubes were used; an 8.5mL Paxgene (Qiagen) tube for DNA extraction (M1, M2, CB), a 4mL lavender top k2EDTA tube for maternal plasma metabolites at M1 and M2 and a 7 mL lavender top k3EDTA tube for umbilical cord (CB) plasma metabolites. Plasma was centrifuged at 2400 RPM and 4°C for 30 minutes, aliquoted with glass pipettes, stored in 4mL clear screw thread glass vials and with F217 (foam) lined polypropylene caps, and stored in a -80°C freezer until metabolomics analyses were conducted.

Rationale for Directed Metabolomics Measures. Since this is the first study to compare metabolomics measures in pregnant women across trimesters, and to examine similarities between maternal and fetal metabolomics profiles, directed metabolomics provide more interpretable, early data compared to untargeted metabolomics, which would provide a more comprehensive measure of all metabolites present, but would include many as yet unidentified metabolites. Transfer of maternal metabolites across the placenta must occur in order for healthy fetal growth and development. Acylcarnitines (ACs) are known to diffuse across cell membranes (1), amino acids (AAs) are transported via carrier proteins (2, 3), while recent evidence suggests free fatty acids (FAs) may be taken up by the placenta in vesicles (1, 4). By measuring all three classes of metabolites, the diffusion, carrier-protein transport, and vesicle uptake mechanisms are all included in this analysis of maternal-infant metabolomes. Maternal metabolism and control of many nutrients, like glucose (5), calcium (6), and long-chain fatty acids (LCFA) (4) change over the course of the pregnancy based on fetal needs and specific tissue development periods. Thus, measuring circulating metabolites in 1st trimester (M1) and at delivery (M2) provide insight into temporal changes.

Directed Metabolomics Quality Control

Internal standards were added to each tube prior to any sample preparation, which allows for assessment of sample loss and quality degradation during processing. Additionally, sample blanks, process blanks and blank blanks were interspersed at multiple intervals throughout the test samples to measure instrument drift and to act as negative controls. The Agilent 7890A-5975C gas chromatography-mass spectrometry (GC-MS) used to quantify AAs and FFAs, injected one µL of each sample onto a Silex column (30m x 250µM x 0.25µM) and eluted at 1mL/minute. The Agilent 1200 LC / 6530 qTOF LC-MS used to quantify ACs used a reverse-phase column (1.8µ, 50 mm x 2.1mm). Both positive and negative mode ion detection were used with 7 µL and 12µL injection volumes, respectively. The column temperature was constant at 40°C with a flow rate of 0.35mL/minute.

DNA Isolation and Bisulfite Conversion*.*

Genomic DNA was extracted from leukocytes in PAXgene blood collection tubes using the PAXgene Blood DNA Kit (PreAnalytiX/Qiagen, Hombrechtikon, Switzerland). DNA extraction and isolation protocols followed manufacturer's instructions. DNA quantity and quality were measured with a ND2000 spectrophotometer (NanoDrop Technology, Wilmington, DL). Genomic DNA for all samples was bisulfite converted to generate methylation dependent genome-wide changes in DNA sequence. Bisulfite treatment converts unmethylated cytosines to uracil, which subsequent polymerase chain reaction (PCR) converts to a thymine nucleotide. Methylated cytosines remain unchanged, thus a distinction can be made between cytosines and thymines at the loci of interest. Bisulfite conversion utilizes about 1 µg of genomic DNA per blood sample in an EpiTect Bisulfite Kit (Qiagen Inc., Valencia, CA) and QIAcube® purification system.

Pyrosequencing Quality Control
All samples were run in duplicate with methylation standards: highly and lowly methylated human genomic control standards for LUMA (EpigenDx, Hopkinton, MA) and Epitect 100% and 0% methylated controls (Qiagen) for all other gene loci. Pyrosequencing was run in two batches due to timing of participant consent in the study. There was no statistical difference in mean methylation status of candidate genes between batches. Global methylation, LUMA and LINE1, varied by batch ($p < 0.001$) but birth gender and birth weight were similar within the batches. Thus, raw methylation values were used in bivariate analyses reported in this manuscript, but future multivariable analyses utilizing this data should also adjust for batch. Percent methylation at each CpG site was averaged between duplicate runs for a final measure used in analyses. Mean methylation at all measured CpG sites in gene promoters was calculated and compared to patterns of methylation at individual CpG sites within each gene. There was significant variability between methylation status at each CpG site within an amplicon and they correlated differently with metabolites and methylation at other genes of interest; therefore, all analyses were run including individual CpG sites for each candidate loci.

LUMA Assay
The Luminometric Methylation Assay (LUMA) uses restriction enzymes to digest genomic DNA and detects methylation at 5'-CCGG-3' sequences across the genome. A detailed description of LUMA analysis via pyrosequencing has been described previously (7, 26). Methylation insensitive *MspI* (Biolabs, Ipswich, MA) and methylation sensitive *HpaII* (Biolabs) enzymes are added to 300 ng of genomic DNA with internal standard *EcoR1* (Biolabs) enzyme and 10x Buffer Tango™ with BSA (Fermentas, Grand Island, NY) for differential digestion. To facilitate digestion, samples were incubated for four hours at 37°C. PyroMarkMD software is run with the following nucleotide dispensation: GTGTCACATGTGTG. Global DNA methylation is calculated by the ratio of normalized product signal between methylation sensitive and insensitive digestions from the same sample: 1-[(*HpaII*/*EcoR1*) / (*MspI*/*EcoR1*)] x 100.

Statistical Analyses
Repeated Maternal Measures. Plasma metabolites were right skewed, so median and interquartile range (IQR) were used to compare M1 and M2 levels, and significance was tested via non-parametric Wilcoxon Signed Rank test (**Table S1**). Methylation status of CpG sites in the promoter region of global and candidate gene loci of interest were normally distributed. Thus, comparisons were made between mean and standard deviation at M1 and M2; tested via Paired T-test (**Table S3**). Significance for both tests of central tendency was determined by a p-value < 0.05.

Bivariate Correlation Analysis. Multiple levels of statistical analyses were performed to fully understand the complex relationships between metabolites and DNA methylation. Simple bivariate correlations (Kendall's Tau) were used to compare metabolome and methylome in the three sample sets (**Tables S2 and S4**) and were calculated to compare each metabolite and CpG site (**Figure S1**). Many bivariate correlations were significant, however, imprinted gene loci, *IGF2* and *H19* had strikingly few significant correlations in this initial analysis (**Figure S2**). Thus, based on this analysis alone, it appears that global methylation and non-imprinted gene methylation are associated with maternal metabolites but that imprinted gene loci are not impacted by these factors.

False Discovery Rate Correction. To correct for multiple testing among metabolites in related pathways and methylation sites within the same gene loci, the Benjamini-Hochberg method was used to calculate adjusted p-values for false discovery rate (FDR) (27). Statistical significance was determined by resulting adjusted p-values < 0.05. These analyses were used to assess the correlations between maternal and infant methylome and metabolome in the 1st trimester and at delivery. Correcting for multiplicity of test

decreased the number of significant associations (**Table S3 and S4**). Using FDR for multiple testing retains more power than the commonly used Bonferroni method.

References

1. Klaassen CD, Aleksunes LM (2014) Xenobiotic , Bile Acid , and Cholesterol Transporters : 62(1):1–96.
2. Jansson T, Aye ILMH, Goberdhan DCI (2012) The emerging role of mTORC1 signaling in placental nutrient-sensing. *Placenta* 33 Suppl 2:e23–9.
3. Larqué E, Ruiz-Palacios M, Koletzko B (2013) Placental regulation of fetal nutrient supply. *Curr Opin Clin Nutr Metab Care* 16(3):292–7.
4. Gil-Sánchez A, Koletzko B, Larqué E (2012) Current understanding of placental fatty acid transport. *Curr Opin Clin Nutr Metab Care* 15(3):265–72.
5. Visiedo F, et al. (2013) High glucose levels reduce fatty acid oxidation and increase triglyceride accumulation in human placenta. *Am J Physiol Endocrinol Metab* 305(2):E205–12.
6. Heringhausen J, Montgomery KS (2005) Continuing education module-maternal calcium intake and metabolism during pregnancy and lactation. *J Perinat Educ* 14(1):52–7.
7. Karimi M, Johansson S, Ekstrom TJ (2006) Using LUMA: a Luminometric-based assay for global DNA-methylation. *Epigenetics* 1:45–48.
8. Lind L, et al. (2013) Global DNA hypermethylation is associated with high serum levels of persistent organic pollutants in an elderly population. *Environ Int* 59:456–461.
9. Yang A, et al. (2004) A simple method for estimating global DNA methylation using bisulfite PCR of repetitive DNA elements. *Nucleic Acids Res* 32:e38.
10. Florea A (2013) for the Assessment of Epigenetic Active Environmental or Clinical Relevant Chemicals. 2013.
11. Price EM, Robinson WP (2012) Response to: "Response to Different measures of “genome-wide” DNA methylation exhibit unique properties in placental and somatic tissues". *Epigenetics* 7(8):965.
12. Wutz a, et al. (1997) Imprinted expression of the Igf2r gene depends on an intronic CpG island. *Nature* 389(6652):745–9.
13. Thorvaldsen JL, Fedoriw AM, Nguyen S, Bartolomei MS (2006) Developmental Profile of H19 Differentially Methylated Domain (DMD) Deletion Alleles Reveals Multiple Roles of the DMD in Regulating Allelic Expression and DNA Methylation at the Imprinted H19 / Igf2 Locus. *Mol Cell Biol* 26(4):1245.
14. Hackett J, Ssurani M (2013) DNA methylation dynamic during the mammalian life cycle. *Philos Trans R Soc London, Biol Sci* 368(1609):20110328.
15. Kwong WY, et al. (2006) Imprinted gene expression in the rat embryo-fetal axis is altered in response to periconceptional maternal low protein diet. *Reproduction* 132(2):265–77.
16. Gong L, Pan Y-X, Chen H (2010) Gestational low protein diet in the rat mediates Igf2 gene expression in male offspring via altered hepatic DNA methylation. *Epigenetics* 5(7):619–626.
17. Hoyo C, et al. (2011) Methylation variation at IGF2 differentially methylated regions and maternal folic acid use before and during pregnancy. *Epigenetics* 6(7):928–936.

18. Perkins E, et al. (2012) Insulin-like growth factor 2/H19 methylation at birth and risk of overweight and obesity in children. *J Pediatr* 161(1):31–9.

19. Lui JC, Finkielstain GP, Barnes KM, Baron J (2008) An imprinted gene network that controls mammalian somatic growth is down-regulated during postnatal growth deceleration in multiple organs. 1103:189–196.

20. Schroeder JW, et al. (2011) Neonatal DNA methylation patterns associate with gestational age. *Epigenetics* 6(12):1498–504.

21. Cordero P, Gomez-Uriz a M, Campion J, Milagro FI, Martinez J a (2013) Dietary supplementation with methyl donors reduces fatty liver and modifies the fatty acid synthase DNA methylation profile in rats fed an obesogenic diet. *Genes Nutr* 8(1):105–13.

22. Mori H, Matsuda KI, Tsukahara S, Kawata M (2010) Intrauterine position affects estrogen receptor α expression in the ventromedial nucleus of the hypothalamus via promoter DNA methylation. *Endocrinology* 151(12):5775–81.

23. Sertic J, et al. (2009) Variants of ESR1, APOE, LPL and IL-6 loci in young healthy subjects: association with lipid status and obesity. *BMC Res Notes* 2:203.

24. Rees WD, McNeil CJ, Maloney C a (2008) The Roles of PPARs in the Fetal Origins of Metabolic Health and Disease. *PPAR Res* 2008:459030.

25. Garratt ES, et al. (2013) Tissue-specific 5′ heterogeneity of PPARα transcripts and their differential regulation by leptin. *PLoS One* 8(6):e67483.

26. Karimi M, et al. (2006) LUMA (LUminometric Methylation Assay) - A high throughput method to the analysis of genomic DNA methylation. *Exp Cell Res* 312:1989–1995.

27. Benjamini Y, Hochberg Y (1995) Controlling the False Discovery Rate : a Practical and Powerful Approach to Multiple Testing. *J R Stat Soc* 57(1):289–300.

Table S1. Comparison of Maternal Median Metabolites from M1 to M2 (n=37)

Absolute Metabolite (*nmol/mL*)	Median (IQR)		Wilcoxon Signed Rank Test[*]	p-value[‡]
	Maternal Baseline (M1)	Maternal Delivery (M2)		
		Acylcarnitines		
L-carnitine	24.60 (21.83-28.9)	12.63 (10.43-17.61)	699.00	***$1.02*10^{-10}$***
C2.0	4.34 (3.74-5.52)	4.06 (3.13-5.72)	418.00	0.323
C3.0	2.22 (1.89-3.09)*10^{-1}	1.34 (1.05-1.71)*10^{-1}	666.00	***$8.35*10^{-8}$***
C4.0	14.85 (11.46-19.67)*10^{-2}	9.30 (8.06-12.91)*10^{-2}	642.00	***$1.61*10^{-6}$***
C5.0	5.52 (4.36-6.72)*10^{-2}	4.24 (3.38-5.81)*10^{-2}	532.00	***$5.62*10^{-3}$***
**C5.0dc**	1.49 (1.18-2.58)*10^{-3}	3.64 (2.42-4.70)*10^{-3}	77.00	***$8.16*10^{-6}$***
**C6.0**	8.71 (6.06-14.01)*10^{-3}	16.73 (10.55-20.72)*10^{-3}	179.00	***$8.34*10^{-3}$***
**C8.0**	2.58 (1.22-4.53)*10^{-2}	4.40 (2.72-6.19)*10^{-2}	260.00	0.172
**C8.1**	7.55 (5.96-11.53)*10^{-2}	9.12 (6.54-13.25)*10^{-2}	241.00	0.097
**C10.0**	3.26 (1.80-5.68)*10^{-2}	6.08 (3.17-7.77)*10^{-2}	236.00	0.083
C10.1	4.98 (1.86-94.56)*10^{-2}	4.38 (2.89-192.10)*10^{-2}	283.00	0.309
**C12.0**	1.49 (0.92-2.97)*10^{-2}	2.92 (1.43-4.06)*10^{-2}	201.00	***0.022***
**C12.0OH**	9.62 (6.65-16.53)*10^{-4}	20.31 (13.47-35.92)*10^{-4}	195.00	***0.017***
**C12.1**	6.68 (4.65-11.46)*10^{-3}	23.23 (11.04-40.33)*10^{-3}	59.00	***$7.92*10^{-3}$***
**C14.0**	5.42 (4.63-9.24)*10^{-3}	8.64 (6.08-12.45)*10^{-3}	220.00	***0.047***
**C14.0OH**	7.23 (4.82-10.75)*10^{-4}	11.40 (6.75-15.97)*10^{-4}	206.00	***0.027***
**C14.1**	5.12 (3.15-10.74)*10^{-3}	12.16 (5.84-23.09)*10^{-3}	193.00	***0.016***
**C14.2**	1.70 (1.15-3.03)*10^{-3}	3.43 (2.09-6.74)*10^{-3}	115.00	***0.026***
**C16.0**	4.79 (3.96-6.62)*10^{-2}	4.90 (3.40-6.31)*10^{-2}	370.00	0.571
**C16.0OH**	9.50 (6.16-12.27)*10^{-4}	9.77 (8.60-12.47)*10^{-4}	301.00	0.455
**C16.1**	9.69 (5.16-29.36)*10^{-3}	14.86 (8.42-50.16)*10^{-3}	223.00	0.053
C18.0	2.78 (1.63-4.24)*10^{-2}	1.93 (1.47-3.07)*10^{-2}	556.00	***$1.52*10^{-3}$***
**C18.1**	9.12 (6.52-15.71)*10^{-2}	9.39 (6.73-12.56)*10^{-2}	333.00	0.788
C18.2	3.94 (2.57-5.69)*10^{-2}	3.22 (2.43-4.38)*10^{-2}	433.00	0.225
**C18.2OH**	5.68 (3.80-8.22)*10^{-4}	14.20 (6.98-24.15)*10^{-4}	142.00	***$1.13*10^{-3}$***
C20.0	16.85 (10.45-25.31)*10^{-4}	8.86 (4.95-17.36)*10^{-4}	224.00	***$7.45*10^{-3}$***
C20.1	14.91 (13.04-19.49)*10^{-4}	9.80 (5.94-13.67)*10^{-4}	254.00	***$1.28*10^{-4}$***
C20.2	3.47 (2.89-5.12)*10^{-3}	3.17 (2.32-4.22)*10^{-3}	180.00	0.211
C20.3	2.45 (1.98-3.33)*10^{-3}	1.79 (1.15-2.33)*10^{-3}	213.00	***0.021***
C20.4	13.33 (8.14-16.40)*10^{-4}	8.78 (6.07-13.43)*10^{-4}	201.00	0.056
		Free Fatty Acids		
**14.0**	2.37 (0.83-3.39)	2.72 (1.89-4.67)	215.50	***0.041***
**16.0**	152.70 (113.90-190.10)	241.40 (193.70-331.00)	64.00	***$2.22*10^{-6}$***
**16.1**	4.91 (2.67-6.76)	10.80 (6.90-18.07)	51.00	***$5.14*10^{-7}$***
**18.0**	93.05 (76.58-114.80)	132.60 (104.60-165.60)	135.00	***$7.29*10^{-4}$***
**18.1n7**	6.37 (5.15-11.32)	16.12 (11.79-24.83)	65.00	***$2.47*10^{-6}$***
**18.1n9**	95.51 (70.63-174.10)	247.90 (183.20-383.50)	61.00	***$1.61*10^{-6}$***
**18.2**	63.18 (47.64-94.46)	105.80 (78.26-179.10)	61.00	***$1.61*10^{-6}$***
18.3n6	0.80 (0.00-2.90)	0.53 (0.00-2.22)	239.00	0.120
**18.3n3**	1.00 (0.30-1.51)	1.98 (1.06-3.80)	147.50	***$2.14*10^{-3}$***
**20.0**	1.36 (0.15 – 2.15)	1.98 (1.26-2.85)	174.50	0.059
**20.1**	1.66 (0.44-2.18)	3.88 (2.69-5.67)	57.50	***$1.03*10^{-6}$***
**20.2**	2.35 (1.75-3.44)	3.20 (2.45-4.58)	162.00	***$4.35*10^{-3}$***
**20.3**	3.85 (2.42-4.64)	4.41 (2.85-7.32)	174.00	***0.021***
**20.4**	6.65 (4.41-9.49)	9.12 (6.75-10.87)	176.50	***$8.47*10^{-3}$***
20.5	0.83 (0.00-1.27)	0.83 (0.00-1.59)	274.50	0.617

**22.0**	1.13 (0.00-2.28)	1.75 (0.00-3.42)	146.50	0.202
22.1	0.00 (0.00-0.80)	0.00 (0.00-0.30)	85.50	***0.041***
22.4	2.57 (0.63-3.66)	2.32 (1.52-4.02)	221.50	0.081
22.5	0.15 (0.00-0.58)	0.00 (0.00-0.54)	68.50	0.472
22.6	0.00 (0.00-0.68)	0.00 (0.00-0.60)	75.00	0.663
24.0	0.00 (0.00-2.09)	0.00 (0.00-1.71)	92.50	0.215
24.1	0.00 (0.00-3.43)	0.00 (0.00-3.10)	68.00	0.705
**Sum FFA (nmol)**	478.10 (385.50-669.30)	839.90 (663.20-1254.00)	61.00	$\boldsymbol{8.09*10^{-7}}$
Amino Acids				
**α-amino-isobutyric acid**	8.78 (7.31-10.96)	9.25 (7.44-10.90)	353.00	0.988
**Alanine**	357.70 (324.60-412.60)	373.50 (312.50-455.00)	308.00	0.521
Asparagine	42.53 (36.45-48.97)	37.68 (32.61-42.48)	530.00	$\boldsymbol{6.21*10^{-3}}$
**Aspartic Acid**	4.23 (2.13-7.16)	7.00 (3.81-11.12)	208.00	***0.030***
**Cysteine**	5.10 (3.62-8.45)	7.28 (5.34-10.35)	111.50	$\boldsymbol{7.47*10^{-3}}$
**Glutamic Acid**	34.28 (22.21-60.94)	73.93 (47.62-105.80)	88.00	$\boldsymbol{2.22*10^{-5}}$
Glutamine	373.40 (297.90-456.60)	364.50 (254.00-476.90)	403.00	0.446
Glycine	189.90(137.10-226.70)	159.80 (123.00-199.40)	554.00	$\boldsymbol{1.71*10^{-3}}$
**Histidine**	40.12 (31.78-47.99)	41.22 (32.16-51.66)	286.00	0.331
Isoleucine	51.16 (44.24-62.88)	36.91 (30.20-44.50)	585.50	$\boldsymbol{2.34*10^{-4}}$
Leucine	104.00 (91.60-122.30)	75.01 (68.72-92.45)	611.00	$\boldsymbol{3.13*10^{-5}}$
Lysine	117.40 (106.30-143.50)	110.80 (80.22-144.40)	490.00	***0.036***
Methionine	12.74 (8.83-14.78)	11.46 (6.73-14.92)	377.00	0.709
Ornithine	41.03 (31.42-50.09)	33.56 (24.35-42.24)	511.00	***0.015***
Phenylalanine	47.52 (40.06-52.90)	44.01 (35.40-49.30)	481.50	0.051
**4-OH Proline**	3.89 (3.03-5.15)	5.77 (4.45-7.87)	122.00	$\boldsymbol{3.09*10^{-4}}$
Proline	174.80 (147.10-207.30)	138.50 (120.60-164.60)	590.00	$\boldsymbol{1.63*10^{-4}}$
**Sarcosine**	6.34 (4.10-8.40)	8.35 (6.38-10.07)	193.50	***0.017***
Serine	75.74 (64.22-88.92)	74.66 (60.98-88.83)	420.00	0.305
**Threonine**	95.58 (86.35-113.90)	142.20 (116.60-167.30)	50.00	$\boldsymbol{4.40*10^{-6}}$
Tryptophan	28.62 (23.25-35.92)	18.00 (13.86-28.11)	602.00	$\boldsymbol{5.18*10^{-5}}$
Tyrosine	33.06 (27.09-37.18)	24.86 (19.93-31.24)	562.00	$\boldsymbol{1.06*10^{-3}}$
Valine	221.40 (185.70-264.10)	149.90 (127.70-177.50)	647.00	$\boldsymbol{9.22*10^{-7}}$
**Percent Metabolite (*nmol/mL*)**	**Median (IQR)**		**Wilcoxon Signed Rank Test***	**p-value‡**
	**Maternal Baseline (M1)**	**Maternal Delivery (M2)**		
Free Fatty Acids				
14.0	0.54 (0.22-0.77)	0.40 (0.22-0.62)	-0.919	0.358
16.0	30.24 (27.07-36.49)	28.07 (24.65-31.50)	-3.070	***0.002***
**16.1**	0.90 (0.65-1.19)	1.32 (1.01-1.65)	-3.900	***0.000***
18.0	19.09 (17.08-21.65)	15.02 (12.09-18.41)	-3.704	***0.000***
**18.1n7**	1.44 (1.30-1.79)	1.90 (1.65-2.06)	-3.523	***0.000***
**18.1n9**	20.15 (17.69-27.30)	29.50 (26.01-34.05)	-4.171	***0.000***
18.2	13.13 (10.24-14.81)	12.85 (11.46-15.09)	-0.143	0.886
18.3n6	0.17 (0.00-0.80)	0.08 (0.00-0.36)	-1.932	0.053
**18.3n3**	0.21 (0.03-0.30)	0.22 (0.15-0.36)	-2.057	***0.040***
**20.0**	0.00 (0.00-0.00)	0.25 (0.17-0.36)	-4.918	***0.000***
**20.1**	0.30 (0.08-0.43)	0.47 (0.36-0.51)	-3.613	***0.000***
20.2	0.53 (0.35-0.68)	0.41 (0.30-0.48)	-2.368	***0.018***
20.3	0.79 (0.43-0.99)	0.52 (0.37-0.74)	-2.686	***0.007***
20.4	1.30 (1.04-1.68)	0.98 (0.70-1.27)	-3.492	***0.000***

20.5	0.17 (0.00-0.28)	0.13 (0.00-0.21)	-1.419	0.156
22.0	0.25 (0.00-0.47)	0.22 (0.00-0.47)	-0.319	0.750
22.1	0.00 (0.00-0.23)	0.00 (0.00-0.03)	-2.521	***0.012***
22.4	0.44 (0.13-0.72)	0.27 (0.16-0.43)	-2.077	***0.038***
22.5	0.00 (0.00-0.16)	0.00 (0.00-0.10)	-1.778	0.075
22.6	0.00 (0.00-0.18)	0.00 (0.00-0.12)	-0.059	0.953
24.0	0.00 (0.00-0.52)	0.00 (0.00-0.29)	-2.959	***0.003***
24.1	0.00 (0.00-0.74)	0.00 (0.00-0.36)	-2.534	***0.011***

* Metabolite levels were right skewed so median and Wilcoxon Signed Rank test were used to test significant changes between maternal metabolites in Trimester 1 (M1) and Delivery (M2).
‡ Metabolites that change significantly ($p < 0.05$) from M1 to M2 have ***bolded and italicized*** p-values.
Bolded metabolite names denote those with a median increase from M1 to M2; metabolites that decreased in plasma level from M1 to M2 are in normal, non-bolded text.

Table S2. Non-parametric Correlations between Maternal-Infant Metabolomes

Metabolite	Mother Trimester 1 vs. Delivery (M1 vs. M2) n=37			Mother Trimester 1 vs. Infant Cord Blood (M1 vs. CB) n=32			Mother Delivery vs. Infant Cord Blood (M2 vs. CB) n=32		
	Kendall's Tau (r)	p-value	Adjusted p-value	Kendall's Tau (r)	p-value	Adjusted p-value	Kendall's Tau (r)	p-value	Adjusted p-value
Acylcarnitines									
L-carnitine	0.213	0.065	0.033*	0.290	0.020*	0.015*	0.258	0.039*	0.024*
C2.0	0.243	0.035*	0.022*	0.044	0.736	0.182	0.169	0.180	0.071
C3.0	0.375	0.001***	0.006**	0.073	0.573	0.154	0.044	0.736	0.182
C4.0	0.366	0.001**	0.006**	0.105	0.411	0.122	0.085	0.509	0.142
C5.0	0.219	0.058	0.030*	0.137	0.279	0.095	0.133	0.294	0.097
C5.0DC	0.048	0.687	0.173	0.190	0.132	0.057	0.181	0.150	0.063
C6.0	0.225	0.051	0.028*	0.137	0.280	0.095	0.060	0.641	0.166
C8.0	0.090	0.443	0.129	0.056	0.664	0.170	0.238	0.057	0.030*
C8.1	0.330	0.003**	0.007**	0.254	0.042*	0.025*	0.411	0.001***	0.006**
C10.0	0.186	0.108	0.049*	0.274	0.028*	0.019*	0.335	0.007**	0.008**
C10.1	0.553	0.000***	0.006**	0.653	0.000***	0.006**	0.617	0.000***	0.006**
C12.0	0.162	0.163	0.068	0.149	0.239	0.086	0.190	0.132	0.057
C12.0OH	0.150	0.197	0.075	0.218	0.083	0.040*	0.254	0.042*	0.025*
C12.1	0.159	0.290	0.097	0.252	0.128	0.056	0.201	0.189	0.073
C14.0	-0.159	0.171	0.070	0.149	0.239	0.086	0.117	0.358	0.110
C14.0OH	0.129	0.268	0.094	0.145	0.252	0.089	0.149	0.239	0.086
C14.1	0.171	0.140	0.060	0.004	0.987	0.225	0.250	0.045*	0.027*
C14.2	0.148	0.271	0.095	0.123	0.417	0.123	0.180	0.187	0.073
C16.0	0.117	0.323	0.102	0.109	0.393	0.119	0.153	0.236	0.086
C16.0OH	0.225	0.051	0.028*	0.036	0.784	0.190	0.331	0.007**	0.008**
C16.1	0.411	0.000***	0.006**	0.484	0.000***	0.006**	0.472	0.000***	0.006**
C18.0	0.339	0.003**	0.006**	0.431	0.000***	0.006**	0.407	0.001***	0.006**
C18.1	0.075	0.524	0.145	0.274	0.028*	0.019*	0.302	0.015*	0.012*
C18.2	0.300	0.009**	0.009**	0.210	0.095	0.043*	0.323	0.009**	0.009**
C18.2OH	-0.057	0.631	0.164	0.085	0.509	0.142	0.169	0.180	0.071
C20.0	0.123	0.432	0.127	0.170	0.332	0.104	0.403	0.015*	0.013*
C20.1	0.375	0.012*	0.010*	0.181	0.298	0.097	0.076	0.679	0.173
C20.2	0.107	0.597	0.142	0.123	0.489	0.141	0.333	0.049*	0.028*
C20.3	0.265	0.081	0.039	0.041	0.836	0.197	0.404	0.016*	0.013*
C20.4	0.209	0.172	0.070	0.088	0.629	0.164	0.287	0.093	0.043*
Free Fatty Acids									
14.0	0.260	0.024*	0.016*	0.218	0.080	0.039*	0.404	0.001**	0.006**
16.0	0.039	0.745	0.184	-0.105	0.411	0.122	0.246	0.049*	0.028*
16.1	0.053	0.647	0.166	-0.040	0.760	0.185	0.208	0.095	0.043*
18.0	-0.090	0.443	0.129	-0.073	0.573	0.154	0.133	0.294	0.097
18.1 n-7	0.060	0.601	0.159	0.026	0.833	0.197	0.066	0.593	0.158
18.1 n-9	0.114	0.329	0.104	0.165	0.191	0.073	0.085	0.509	0.142
18.2	0.210	0.069	0.035*	0.194	0.124	0.055	0.016	0.910	0.211
18.3 n-6	0.357	0.004**	0.007**	0.593	0.000***	0.006**	0.489	0.000***	0.006**
18.3 n-3	-0.119	0.274	0.095	0.392	0.003**	0.006**	-0.083	0.519	0.144
20.0	0.256	0.031*	0.020*	0.560	0.000***	0.006**	0.269	0.042*	0.025*
20.1	0.047	0.692	0.173	0.014	0.918	0.212	-0.040	0.760	0.185
20.2	0.363	0.002**	0.006**	0.257	0.039*	0.024*	-0.032	0.795	0.191
20.3	0.297	0.010*	0.010**	0.423	0.001***	0.006**	0.470	0.000***	0.006**
20.4	0.214	0.063	0.033*	0.236	0.058	0.030*	0.234	0.062	0.032*

20.5	0.125	0.306	0.098	0.349	0.011*	0.010*	0.137	0.307	0.098
22.0	0.213	0.085	0.041*	0.547	0.000***	0.006**	0.165	0.228	0.085
22.1	0.706	0.000***	0.006**	0.574	0.000***	0.006**	0.763	0.000***	0.006**
22.4	0.270	0.021*	0.015*	0.320	0.012*	0.010*	0.382	0.003**	0.096**
22.5	0.567	0.000***	0.006**	0.629	0.000***	0.006**	0.545	0.000***	0.006**
22.6	0.584	0.000***	0.006**	0.637	0.000***	0.006***	0.621	0.000***	0.006**
24.0	0.652	0.000***	0.006**	0.580	0.000***	0.006**	0.750	0.000***	0.006**
24.1	0.754	0.000***	0.006**	0.776	0.000***	0.006**	0.621	0.000***	0.006**
FFA Sum (Nmol)	0.030	0.803	0.192	-0.030	0.818	0.195	0.077	0.551	0.150
Amino Acids									
AlphaAminoisobutyricAcid	0.250	0.030*	0.020*	0.077	0.538	0.147	0.457	0.000***	0.006*
Alanine	0.511	0.000***	0.006**	-0.012	0.936	0.215	0.169	0.180	0.071
Asparagine	0.280	0.015*	0.012*	0.279	0.025*	0.017*	0.170	0.173	0.070
Aspartic Acid	0.135	0.246	0.088	0.226	0.072	0.036*	0.331	0.007**	0.008*
Cysteine	0.363	0.004**	0.007**	0.316	0.021**	0.015*	0.440	0.001**	0.006**
Glutamic Acid	0.108	0.356	0.110	0.052	0.688	0.173	0.371	0.003**	0.006**
Glutamine	0.264	0.021*	0.015*	-0.008	0.962	0.220	0.065	0.618	0.162
Glycine	0.450	0.000***	0.006**	0.565	0.000***	0.006**	0.427	0.000***	0.006**
Histidine	0.348	0.002**	0.006**	0.383	0.002**	0.006**	0.355	0.004**	0.007**
4-Hydroxyproline	0.123	0.283	0.096	0.129	0.299	0.097	0.016	0.897	0.210
Isoleucine	0.065	0.534	0.154	0.083	0.506	0.142	0.135	0.277	0.095
Leucine	0.108	0.356	0.110	0.016	0.910	0.211	0.246	0.049*	0.028*
Lysine	0.270	0.018*	0.014*	-0.081	0.530	0.146	0.286	0.021*	0.015*
Methionine	0.120	0.295	0.097	0.212	0.089	0.042*	0.162	0.194	0.074
Ornithine	0.162	0.163	0.068	-0.069	0.595	0.158	0.145	0.252	0.089
Phenylalanine	0.194	0.092	0.043*	0.129	0.310	0.099	0.153	0.226	0.085
Proline	0.204	0.077	0.038*	0.105	0.411	0.122	0.198	0.116	0.052
Sarcosine	0.323	0.005**	0.007**	-0.117	0.359	0.110	-0.026	0.838	0.197
Serine	0.237	0.039*	0.024*	0.113	0.375	0.114	0.242	0.053	0.029*
Threonine	0.153	0.188	0.073	0.036	0.785	0.190	0.258	0.039*	0.024*
Tryptophan	0.365	0.001**	0.006**	0.373	0.003**	0.006**	0.318	0.012*	0.010*
Tyrosine	0.260	0.024*	0.016*	0.085	0.509	0.142	0.052	0.688	0.173
Valine	0.182	0.113	0.051	0.149	0.239	0.086	0.238	0.057	0.030*

Nonparametric analysis of metabolites across sample time points analyzed by Kendall's Tau correlations. The Benjamini-Hochberg method was used to calculate adjusted p-values, to correct for false discovery rate: *$p \leq 0.05$, **$p \leq 0.01$, ***$p \leq 0.001$. Values were rounded to three decimal points for the table, but significance is assigned by pre-rounded values.

Table S3. Comparison of Maternal Mean Methylation from M1 to M2 (n=37)

Gene Promoter (% methylated)	Mean (SD)		Paired T-test*	p-value‡	95% CI
	Maternal Baseline (M1)	Maternal Delivery (M2)			
LUMA	85.29 (10.61)	86.91 (9.14)	-2.71	***0.010***	***(-2.90, -0.42)***
LINE1_Mean	75.06 (3.26)	75.41 (4.05)	-0.40	0.471	(-1.23, 0.58)
LINE1_Site1	77.27 (3.50)	77.54 (4.15)	-0.22	0.694	(-1.74, 1.17)
LINE1_Site2	77.25 (4.07)	78.16 (4.58)	-1.59	0.120	(-1.85, 0.22)
LINE1_Site3	74.07 (5.06)	74.81 (6.96)	-0.96	0.341	(-2.31, 0.82)
LINE1_Site4	70.82 (4.28)	71.21 (2.91)	-0.67	0.510	(-1.61, 0.82)
*IGF2*_Mean	54.74 (2.89)	55.17 (2.80)	-0.90	0.374	(-1.12, 0.43)
*IGF2*_Site1	52.03 (3.97)	52.97 (3.80)	-1.21	0.234	(-2.16, 0.55)
*IGF2*_Site2	58.08 (2.99)	58.27 (2.94)	-0.26	0.793	(-1.10, 0.84)
*IGF2*_Site3	54.21 (3.08)	54.22 (3.25)	-0.23	0.816	(-0.93, 0.74)
*H19*_Mean	59.75 (3.79)	60.33 (3.56)	-0.79	0.436	(-1.15, 0.51)
*H19*_Site1	61.32 (3.70)	62.20 (3.56)	-1.44	0.158	(-1.93, 0.33)
*H19*_Site2	59.40 (3.58)	60.05 (3.60)	-0.30	0.767	(-0.98, 0.73)
*H19*_Site3	60.28 (4.02)	61.05 (3.92)	-0.86	0.398	(-1.39, 0.56)
*H19*_Site4	57.86 (3.79)	60.33 (3.56)	-0.18	0.856	(-1.27, 1.06)
*ESR1*_Mean	2.97 (0.85)	2.48 (0.59)	3.49	***0.001***	***(0.22, 0.82)***
*ESR1*_Site1	2.47 (0.99)	2.11 (0.79)	2.89	***0.007***	***(0.11, 0.63)***
*ESR1*_Site2	2.54 (0.82)	1.96 (0.75)	3.75	***0.001***	***(0.28, 0.94)***
*ESR1*_Site3	3.91 (1.20)	3.37 (0.94)	2.82	***0.008***	***(0.16, 1.01)***
*PPARα*_Mean	0.97 (0.41)	0.90 (0.33)	0.95	0.350	(-0.08, 0.23)
*PPARα*_Site1	0.57 (0.37)	0.50 (0.35)	1.15	0.258	(-0.05, 0.18)
*PPARα*_Site2	0.86 (0.41)	0.82 (0.49)	0.54	0.585	(-0.13, 0.23)
*PPARα*_Site3	1.38 (0.48)	1.39 (0.51)	-0.10	0.989	(-0.14, 0.14)

Percent DNA methylation was normally distributed at all loci investigated so mean, standard error of the mean, and paired T-test were used to test significant changes between maternal methylation at Trimester 1 (M1) and Delivery (M2). ‡ CpG sites whose methylation status changed significantly ($p < 0.05$) from M1 to M2 have ***bolded and italicized*** p-values and 95% CI.

Table S4. Non-parametric Correlations between Maternal-Infant Methylomes

Gene Locus	Mother Trimester 1 vs. Delivery (M1 vs. M2) n=37			Mother Trimester 1 vs. Infant Cord Blood (M1 vs. CB) n=32			Mother Delivery vs. Infant Cord Blood (M2 vs. CB) n=32		
	Kendall's Tau (r)	p-value	Adjusted p-value	Kendall's Tau (r)	p-value	Adjusted p-value	Kendall's Tau (r)	p-value	Adjusted p-value
Global Methylation									
LUMA average	0.562	0.000***	0.000***	0.424	0.001**	0.002**	0.655	0.000***	0.000***
LINE1_Mean	0.523	0.000***	0.000***	0.407	0.001**	0.002**	0.508	0.000***	0.000***
LINE1_Site1	0.263	0.022*	0.032*	0.242	0.053	0.064	0.301	0.016*	0.024*
LINE1_Site2	0.523	0.000***	0.000***	0.434	0.000***	0.001**	0.478	0.000***	0.000***
LINE1_Site3	0.505	0.000***	0.000***	0.498	0.000***	0.000***	0.547	0.000***	0.000***
LINE1_Site4	0.471	0.000***	0.000***	0.440	0.000**	0.001**	0.252	0.043*	0.053
Candidate Gene Methylation – Imprinted Genes									
*IGF2*_Mean	0.553	0.000***	0.000***	0.184	0.140	0.158	0.192	0.123	0.142
*IGF2*_Site1	0.463	0.000***	0.000***	-0.069	0.581	0.608	-0.145	0.252	0.276
*IGF2*_Site2	0.453	0.000***	0.000***	0.097	0.458	0.486	-0.049	0.711	0.732
*IGF2*_Site3	0.478	0.000***	0.000***	0.421	0.001**	0.002**	0.290	0.022*	0.032*
*H19*_Mean	0.587	0.000***	0.000***	0.399	0.001**	0.002**	0.289	0.020*	0.031*
*H19*_Site1	0.381	0.001**	0.002**	0.276	0.026*	0.036*	0.018	0.884	0.884
*H19*_Site2	0.446	0.000***	0.000***	0.283	0.023*	0.032*	0.192	0.123	0.142
*H19*_Site3	0.531	0.000***	0.000***	0.327	0.009**	0.014*	0.131	0.292	0.315
*H19*_Site4	0.624	0.000***	0.000***	0.446	0.000***	0.001**	0.424	0.001**	0.002**
Candidate Gene Methylation – Non-Imprinted Genes									
*ESR1*_Mean	0.341	0.004**	0.007**	0.377	0.003**	0.006**	0.244	0.050*	0.061
*ESR1*_Site1	0.506	0.000***	0.000***	0.554	0.000***	0.000***	0.566	0.000***	0.000***
*ESR1*_Site2	0.377	0.001**	0.002**	0.020	0.878	0.884	-0.178	0.161	0.179
*ESR1*_Site3	0.340	0.004**	0.007**	0.356	0.005**	0.009**	0.272	0.029*	0.038*
*PPARα*_Mean	0.209	0.069	0.082	0.327	0.009**	0.014*	0.304	0.015*	0.023*
*PPARα*_Site1	0.392	0.001***	0.002**	0.413	0.001**	0.003**	0.522	0.000***	0.000***
*PPARα*_Site2	0.245	0.034*	0.043*	0.473	0.000***	0.000***	0.286	0.023*	0.032*
*PPARα*_Site3	0.329	0.005**	0.009**	0.267	0.032*	0.042*	0.465	0.000***	0.001**

Nonparametric analysis of metabolites across sample time points analyzed by Kendall's Tau correlations. The Benjamini-Hochberg method was used to calculate adjusted p-values, to correct for false discovery rate: *$p \leq 0.05$, **$p \leq 0.01$, ***$p \leq 0.001$.

Table S5. Candidate Loci PCR and Pyrosequencing Parameters

DNA Methylation Regions	Sequence to Analyze	PCR Primers	PCR Cycling Parameters	Sequencing Primer	Volume of PCR Product Used in Pyrosequencing
LINE1	TT**C**GTGGTG**C**<br>GT**C**GTTTTTT<br>AAGT**C**GGTTT<br>GAAAAG	Forward:<br>5'-TTGGTTAGGTGTGGGATATAGTT-3'<br><br>Reverse:<br>5'-CAAAAAATCAAAAAATTCCCTTTCC-3'	95ºC for 14.5 min<br>95ºC for 30 sec<br>58ºC for 30 sec<br>72ºC for 30 sec<br>for 45 cycles<br>4ºC hold	5'-AGGTGTGGATATAGT-3'	10 µL
IGF2	AGTATAGTTA<br>**C**GT**C**GTTTTT<br>TATTGGTTT**C**<br>GTTAAGTAGA	Forward:<br>5'-GGAGGGGGTTTATTTTTTTAGGAAG-3'<br><br>Reverse:<br>5'-AACCCCAACAAAAACCACTAAACAC-3;	95ºC for 15 min<br>[94ºC x 30s, 70ºC x 30s, 72ºC x 30s] x 5 cycles<br>[94ºC x 30s, 68ºC x 30s, 72ºC x 30s] x 5 cycles<br>[94ºC x 30s, 66ºC x 30s, 72ºC x 30s] x 42 cycles<br>72ºC for 5 min<br>4ºC hold	5'-GGGGTTTATTTTTTTAGGA-3'	10µL
H19	GGT**C**G**C**G**C**G<br>G**C**GGTAGTGT<br>AGGTTTATAT<br>ATTATAGTT	Forward:<br>5'-TTTGTTGATTTTATTAAGGGAG-3'<br><br>Reverse:<br>5'-CTATAAATAAACCCCAACCAAAC-3'	95ºC for 15 min<br>[94ºC x 30s, 64ºC x 30s, 72ºC x 30s] x 5 cycles<br>[94ºC x 30s, 61ºC x 30s, 72ºC x 30s] x 5 cycles<br>[94ºC x 30s, 58ºC x 30s, 72ºC x 30s] x 45 cycles<br>72ºC for 5 min<br>4ºC hold	5'-GTGTGGAATTAGAAGT-3'	10 µL
ESR1	TTT**C**GTG**C**GT<br>TTT**C**GGT**C**GT<br>GAAATTTAGT<br>TTT	Forward:<br>5'-GGGGTATATAAGGTAGTATATTAGAGA-3'<br><br>Reverse:<br>5'CAACTTCCCTAAACTTTACTTTACTTAT-3'	95ºC for 15 min<br>95ºC for 30 sec<br>59ºC for 30 sec<br>72ºC for 30 sec<br>for 45 cycles<br>4ºC hold	5'-TTTTTGGGTTATTTTTAGTAGAT-3'	10 µL
PPARα	**C**GTAGGGTGG<br>GAGG**C**GGCC<br>**C**GGGA	Used pre-optimized Qiagen assay (PM00082635) - Primer sequences are proprietary, therefore not available	95ºC for 15 min<br>95ºC for 30 sec<br>62ºC for 30 sec<br>72ºC for 30 sec<br>for 40 cycles<br>4ºC hold	Used pre-optimized Qiagen assay (PM00082635). Primer sequences are proprietary, therefore not available	10 µL

A. **B.**

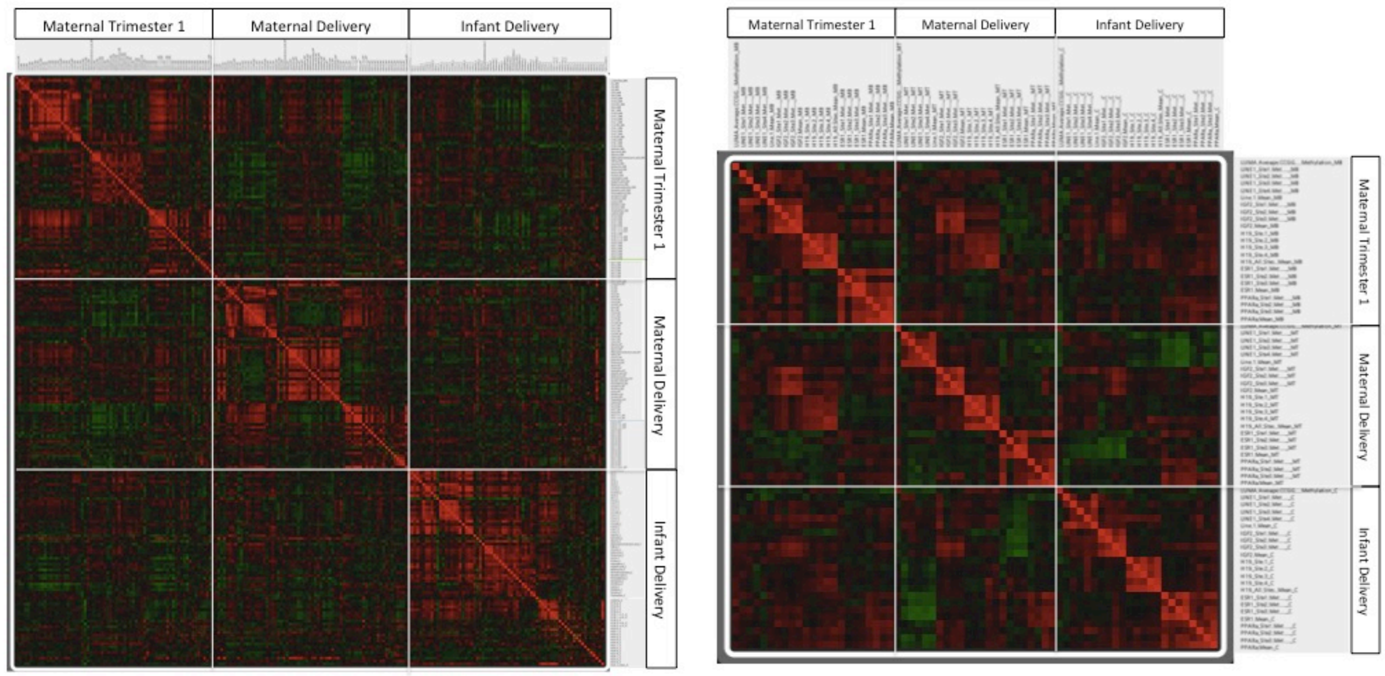

Fig S1. Descriptive Comparison of Maternal-Infant Metabolome and Epigenome across Pregnancy**.** Heatmaps illustrate the Kendall's tau correlation coefficients between blood samples from mother in 1st trimester, delivery, and infant umbilical cord blood for (A) targeted metabolomics (acylcarnitines, fatty acids, and amino acids) and (B) DNA methylation at CpG sites of global (LUMA, LINE1), imprinted (*IGF2,* H19), and non-imprinted (*ESR1, PPARα*) gene loci.

A. Maternal 1st Trimester Metabolites and Infant DNA Methylation Sites

B. Maternal Delivery Metabolites and Infant DNA Methylation Sites

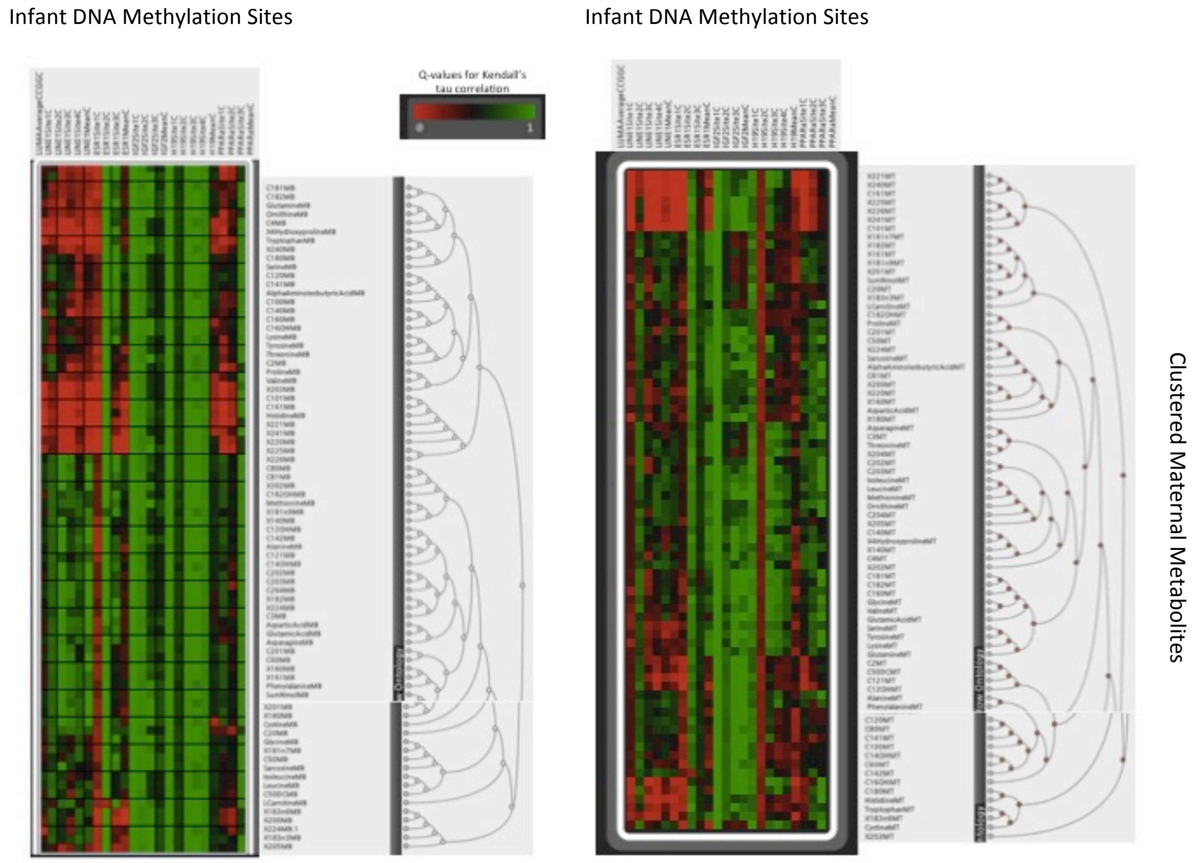

Fig S2. Correlations between CpG Site-specific Infant DNA Methylation and Maternal Metabolites at (A) 1st trimester (M1) and delivery (M2) and in umbilical cord blood (CB). FDR-corrected Kendall's tau correlations (q-values) are presented. Significant correlations (<0.05) are red; non-significant correlations with q-values approaching one are green
